# Topical Tacrolimus in Anterior Segment Disorders in Ophthalmology: A Review

**DOI:** 10.22336/rjo.2024.19

**Published:** 2024

**Authors:** Sultan Homoud Alrashidi

**Affiliations:** * Department of Ophthalmology, College of Medicine, Qassim University, Buraidah, Saudi Arabia

**Keywords:** allergic eye diseases, dry eye, corneal graft, ocular inflammatory conditions, immunosuppressant, topical tacrolimus

## Abstract

The purpose of this study is to emphasize topical tacrolimus’s role in treating anterior segment diseases in ophthalmology. The present study analyzed research papers and publications from international databases, including Pubmed, MedLine, Google Scholar, Cochrane Library, Embase, China National Knowledge Infrastructure (CNKI), and Scopus to highlight the significance and advantages of topical application of tacrolimus and its efficacy in treating allergic eye disorders, immune-mediated diseases, and other ocular surface disorders. Tacrolimus and cyclosporine are the two most commonly used topical immunosuppressants in ophthalmology. Tacrolimus is a selective calcineurin inhibitor administered for the prevention and treatment of allograft rejection in solid organ transplant recipients and has a similar mechanism of action to cyclosporine. Management of immune-mediated inflammatory anterior segment requires intense immunosuppression and studies have shown that tacrolimus is ten to hundred times more effective than cyclosporine.

**Abbreviations:** IL-2 = interleukin-2, FDA = Food and Drug Administration Agency, GvHD = graft versus host disease, (Ig)E = immunoglobulin E, SAC = seasonal conjunctivitis, PAC = perennial allergic conjunctivitis, VKC = vernal keratoconjunctivitis, AKC = allergic keratoconjunctivitis, GPC = giant papillary conjunctivitis, PKC = phyctenular keratoconjunctivitis, DED = dry eye disease, TBUT = tear break up time

## Introduction

***History of Tacrolimus***

Tacrolimus (FK506) is a 23-membered cyclic macrolide lactone with immunomodulatory function derived from the bacteria Streptomyces tsukubaensis in 1984 [**1**]. The generic name of tacrolimus is “Tsukuba macrolide immunosuppressant”. Due to its strong immunosuppressive effects, initial oral and parenteral formulations of tacrolimus have been approved to prevent rejection after organ transplantation. However, it is now clinically used worldwide. Researchers from Chiba University in Japan demonstrated the immunosuppressive effect of tacrolimus at the 11th World Society of Transplantation Congress in Helsinki in 1986. Since then, tacrolimus has been used in various clinical trials to reduce immune function and the risk of post-transplant rejection. Therefore, this drug was initially approved for treating atopic dermatitis in Japan in 1990, followed by the United States in 2000 and Europe in 2001 [**2**].

***Mechanism of action***

Tacrolimus is an immunomodulatory macrolide that becomes biologically active when bound to immunophilins, inhibiting calcineurin activity, and blocking interleukin-2 (IL-2) production and T cell activation. Furthermore, it inhibits the release of histamine from mast cells and other inflammatory cytokines such as IL-3, IL-4, IL-5, IL-8, interferon-gamma, and tumor necrosis factor-alpha, thereby suppressing immune-mediated responses. Therefore, it is an effective immunosuppressant in vivo and in vitro [**3**,**4**].

Numerous clinical trials assessing various formulations and dosages of tacrolimus, for the management of anterior segment inflammatory diseases, were conducted worldwide to study the immunosuppressive properties of tacrolimus for ocular use [**5**-**12**]. Topical tacrolimus (Protopic® ointment 0.1%) is the only drug approved for the treatment of atopic dermatitis by the Food and Drug Administration Agency (FDA) in 1999 and Protopic® ointment 0.03% was approved for pediatric use in 2003 [**13**]. However, it has been effectively used to treat psoriasis, allergic contact dermatitis, graft versus host disease (GvHD), seborrheic dermatitis, chronic actinic dermatitis, allergic asthma, rhinitis, conjunctivitis, and vitiligo [**7**].

In ophthalmology, off-label usage of topical tacrolimus 0.03% is safe and effective in treating anterior segment inflammatory diseases [**9**-**12**]. Tacrolimus and cyclosporine are the two immunosuppressants that are most frequently used topically. Tacrolimus functions analogous to cyclosporine A, although it is 50-100 times more effective and less likely to cause lipid abnormalities and systemic hypertension. In 1989, Kobayashi showed the initial evidence that tacrolimus hydrate (FK506) suppressed corneal graft rejection in rabbits [**14**]. Because of its ability to decrease T-lymphocyte-mediated reactions and its proven effectiveness in treating immune-mediated conditions such as uveitis, ocular inflammation, ocular allergies, post-corneal graft rejection, ocular GvHD, and ocular cicatricial pemphigoid, FK506 has consequently drawn special attention in the field of ophthalmology [**15**-**21**]. The dosage, formulation, and frequency of usage of tacrolimus depend on the nature and severity of the disease [**22**]. This review aims to focus on the available studies in the literature on the successful use of tacrolimus in allergic eye diseases and various T-cell-related ocular diseases.

## Review

A literature review was performed to extract relevant information from databases like Pubmed, MedLine, Google Scholar, Cochrane Library, Embase, China National Knowledge Infrastructure (CNKI), and Scopus. The keywords used for extracting the articles such as “tacrolimus or FK506”, “topical tacrolimus”, “allergic eye disorders”, “conjunctivitis”, “dry eye disease”, “corneal graft”, “keratitis”, “keratoconjunctivitis”, and “anterior segment inflammatory disease” were combined using Boolean logic operations. Titles, abstracts, and full-text articles were screened and scrutinized for their information. Relevant studies and their bibliographies on the efficacy and safety of topical tacrolimus in anterior inflammatory disorders were collected, organized, and classified according to indications for use.

## Topical tacrolimus for allergic eye diseases

“Ocular Allergic Disorders” refers to conditions that affect the eyelids and conjunctiva with or without corneal involvement and can be part of systemic or localized allergies. In 1819, John Bostock reported the first signs of an allergic eye disorder in a 46-year-old man, who had red, itchy, and watery eyes from June to August each year [**23**]. Most ocular allergies are due to allergen-induced, immunoglobulin (Ig)E- and non-IgE-mediated reactions [**24**]. The classification of allergic eye diseases includes different clinical forms and can be divided into two main categories: the most common seasonal conjunctivitis (SAC) and perennial allergic conjunctivitis (PAC). The second category includes the less common severe persistent eye allergies namely vernal keratoconjunctivitis (VKC) and allergic keratoconjunctivitis (AKC) [**24**].

Topical corticosteroids are useful only in the short-term treatment of persistent allergic conjunctivitis, namely AKC and VKC. Furthermore, individuals receiving topical corticosteroid treatment have an increased chance of experiencing serious ocular problems, especially in childhood, when VKC is most common. Since T lymphocytes play an important role in the immunopathology of persistent allergic conjunctivitis, immunomodulatory agents that prevent the triggering of T-cells are likely the appropriate therapeutic agents for these conditions. The use of tacrolimus, an immunomodulator, to treat chronic allergic ocular disorders has become more common in recent years.

Tacrolimus is primarily used to treat persistent allergic conjunctivitis (VKC, AKC) and giant papillary conjunctivitis (GPC). Studies on chronic allergic conjunctivitis management have shown an important role for tacrolimus in significantly reducing symptom incidence by approximately 50% [**25**-**27**]. Yoon CH et al. (2018) evaluated the efficacy of topical tacrolimus 0.03% as non-steroidal maintenance therapy in young patients diagnosed with severe and recurrent steroid-dependent phyctenular keratoconjunctivitis (PKC). During the active phase of PKC, the patients were treated with a combination of tacrolimus and steroids, followed by topical tacrolimus alone after remission. After receiving combination therapy with tacrolimus and steroids for 25.2 ± 16.9 days, all patients showed resolution of their clinical ocular manifestations and improvement in visual acuities. Patients who showed remission received topical tacrolimus 0.03% alone once daily for 8.4 ± 4.7 months, and no recurrence occurred during a follow-up period of 10.6 ± 1.9 months. Two patients were effectively weaned off tacrolimus without experiencing any subsequent relapses [**26**]. Al-Amri et al. (2014) used 0.1% topically administered tacrolimus for 48 months to treat AKC. They were able to achieve disease control and remission without the need for additional medication. No adverse effects, except for a slight burning sensation, were reported [**28**]. Müller et al. (2019) study used 0.03% tacrolimus continuously for 41 months to treat VKC, demonstrating the safety of long-term use by showing no major side effects [**29**].

Both tacrolimus and cyclosporine target CD4+ T cells through calcineurin inhibition, suppressing IL-2 generation, eosinophil recruitment, and the inflammatory cascade. This is how their mechanisms of action are comparable. Another function of tacrolimus is that of a mast cell membrane stabilizer that prevents histamine release and prostaglandins synthesis [**29**]. In studies, tacrolimus has been proven to be more immunosuppressive than cyclosporine. While being 25-100 times more effective than cyclosporin, it has been shown to have fewer adverse effects, such as raising blood pressure or impairing lipid metabolism [**30**].

A review of “topical tacrolimus for allergic eye diseases” by Nir Erdinest et al. (2019) found that a low concentration of topical tacrolimus is effective against VKC and AKC when antihistamines and mast cell stabilizers are often insufficient without concomitant topical corticosteroid treatment. Therefore, in AKC and VKC, a lower concentration of topical tacrolimus (0.02%) is much more effective than cyclosporine A and prevents the development of serious ocular complications of VKC, such as shield ulcers or limbal stem cell deficiency by initiating the early treatment with topical tacrolimus. Due to this, a growing number of eye care providers have started treating severe allergic conjunctivitis using tacrolimus, which, in turn, minimized the need for topical corticosteroids [**31**]. Hence, tacrolimus can be used as an alternative therapy to corticosteroids because it is more effective and has fewer side effects than corticosteroids [**31**,**32**].

Kheirkhah A et al. (2011) used low-dose tacrolimus eye drops (0.005%) in patients with active perennial symptomatic VKC for an average duration of 12.1 ± 5.8 years, who had previously been treated with anti-histamines, mast-cell stabilizers, and topical steroids. After discontinuing all other treatments, ten patients received four daily doses of a topical 0.005% tacrolimus eye drop. Changes in allergic symptoms, signs, and potential consequences were assessed following treatment. The itching was the first symptom that showed a noticeable improvement after receiving tacrolimus treatment; other symptoms including redness, photosensitivity, foreign body sensation, and mucus discharge were also decreased. Conjunctival hyperemia was the first to improve, followed by papillary hypertrophy, giant papillae, limbal hypertrophy, corneal punctate epithelial erosions, and corneal pannus. None of the patients required further medication, including steroids, to relieve their symptoms. Patients were advised to take tacrolimus eye drops consistently for the duration of the follow-up period because discontinuing the eye drops throughout treatment has been linked to the recurrence of patients’ signs and symptoms. Tacrolimus did not cause any ocular complications. This study concluded that the lower dosages of topical tacrolimus eye drops were a safe and successful treatment for steroid-resistant refractory VKC. However, continued administration was required to control the disease [**33**]. 

Fukushima A et al. (2014) conducted a large-scale investigation in a standard clinical environment, evaluating the benefits and risks of tacrolimus eye drop usage in 1436 patients with severe allergic conjunctivitis. Ten clinical signs including palpebral conjunctiva hyperemia and edema, follicles, papillae, giant papillae, bulbar conjunctiva hyperemia, and edema, limbus Trantas dots, swelling, and corneal epithelial signs; and six clinical symptoms including itching, discharge, lacrimation, photophobia, foreign body sensation, and eye pain were evaluated before and after tacrolimus eye drop therapy. A substantial reduction in clinical signs and symptom scores was observed one month after starting treatment with tacrolimus eye drops twice daily [**34**].

Zhao M et al. (2022) conducted a systematic review of the clinical effectiveness of tacrolimus in VKC. From the total of 177 papers retrieved, five research papers were finally chosen, and 203 samples were analyzed, all of which were VKC cases treated with tacrolimus. The concentrations of topical tacrolimus used in the included studies were 0.1%, 0.005%, and 0.03%. According to the review’s results, tacrolimus was beneficial for allergic conjunctivitis and an effective alternative to corticosteroid therapy in reducing disease activity. Furthermore, it was found that the clinical application of tacrolimus for VKC treatment was therapeutically effective, significantly improving ocular signs while decreasing itching, congestion, tearing, foreign body sensation, and other symptoms. Also, most of the reported adverse ocular side effects were mild [**35**].

In summary, corticosteroid eye drops, topical antiallergic medications, and artificial tears are often used to treat severe allergic eye diseases. However, corticosteroid-sparing medications are mandatory because of their potential adverse effects including cataracts and glaucoma [**36**]. Tacrolimus, a topical immunomodulator is a safe substitute for long-term therapy of allergic eye diseases, with relatively temporary adverse effects and no recurrence of symptoms after the medication discontinuation [**37**].

Corneal infections such as herpetic keratitis, bacterial keratitis, and bacterial corneal ulcers were reported in 0.35% of those who used topical tacrolimus for an extended period, while from 0.84% of AKC patients receiving topical cyclosporine, 0.1% developed infectious corneal complications. These findings indicate that tacrolimus eye drops act analogous to cyclosporine and that long-term usage needs continuous monitoring [**38**].

## Topical tacrolimus for Dry eye

The International Dry Eye Workshop (2007) defines dry eye disease (DED) as “a multifactorial disease of the tear film and ocular surface that results in discomfort, visual disturbance, and tear film instability, with potential damage to the ocular surface, associated with ocular surface inflammation and increased tear film osmolarity” [**39**]. Ocular lubricants with or without preservatives, topical corticosteroid therapy, topical immunosuppressive agents, and punctal occlusion with temporary or permanent plugs are the usual treatments for DED.

The primary causes of clinical symptoms and ocular surface inflammation associated with DED seem to be the activation of CD4 T cells and autoimmune inflammation. Tacrolimus suppresses the immune response by blocking T-lymphocytes and inhibiting other inflammatory cytokines. Therefore, tear production can be increased by reducing inflammation by inhibiting inflammatory cytokines and T-cell activation in the lacrimal gland and conjunctiva [**40**]. Moscovici et al. (2012) evaluated the overall effects of using 0.3% topical tacrolimus eye drops (vehicle almond oil) twice daily to treat DED. In all patients, evaluation of tear secretion (Schirmer 1 test), tear stability assessment (Tear break up time-TBUT), and ocular surface damage (corneal fluorescein and rose-bengal staining) were performed on one day before starting treatment, and 14, 28, and 90 days after treatment. Significant improvements were observed in ocular damage assessment scores after 14 days of treatment, and further improvements were observed after 28 and 90 days. The tear secretion assessment showed significant improvement after 90 days of treatment, whereas the tear stability using TBUT showed a significant improvement after 28 and 90 days of treatment with 0.03% tacrolimus eye drops [**4**].

Fatima Z et al. (2018) investigated the effectiveness of tacrolimus and cyclosporine for two groups of DED patients. Group 1 patients were treated with tacrolimus eye ointment (0.03%), and group 2 subjects were given cyclosporine eye drops (0.05%) twice a day. After three months, both treatment groups showed significant improvement in Schirmer score and tear break-up time, results were comparable. This study also stated that 0.03% tacrolimus ointment improved DED signs and symptoms in subjects not responding to artificial tear supplements. Also, 0.03% tacrolimus ointment is just as safe and efficient as 0.05% cyclosporine eyedrops. 0.03% tacrolimus cream may be a good therapeutic alternative for patients having an intolerance to local or systemic cyclosporine. Moreover, it can be used instead of cyclosporine, especially if the DED symptoms do not improve as expected [**20**]. Similarly, Moawad P et al. (2022) conducted a comparative study between topical tacrolimus 0.03% eye drops and cyclosporine 0.05% in treating severe DED in two groups that included individuals with Sjogren syndrome. Group A patients were instructed to use tacrolimus 0.03% eyedrops in one eye for six months and placebo eyedrops in the other. Group B patients were treated with cyclosporine 0.05% eye drops in one eye for six months and placebo eyedrops in the other. Patients were evaluated for the Ocular Surface Disease Index at days 0, 90, and 180. For both immunomodulatory eye drops, the findings showed an improvement in patient symptoms and ocular surface staining ratings were similar [**41**].

In summary, the immunosuppressive agent tacrolimus has been demonstrated to be useful in stabilizing the tear film and improving ocular health in DED patients. The patients’ clinical improvement was better than it had been during their steroid medication, and it persisted even after the steroid was stopped. Thus, it seems that topical tacrolimus, despite its drawbacks, might prove to be a useful alternative to topical steroid therapies in DED.

## Use of tacrolimus in corneal transplantation

One of the most common tissue transplant procedures done globally is corneal transplantation. Immunologic allograft rejection is one of the major reasons for corneal transplant failure in individuals with poor prognosis. Low-risk corneal transplant survival is typically guaranteed by topical corticosteroid therapy. Low-risk grafts have a favorable prognosis, with an average rejection rate of 13.5% after two years [**42**]. Based on parameters used to characterize high-risk grafts, failure rates have ranged from 60% to 90% [**34**,**44**]. Animal models of high-risk transplants have shown improved graft survival when immunosuppression is used topically and systemically with cyclosporin and tacrolimus [**32**,**45**-**47**].

The effectiveness and adverse effects of tacrolimus for patients requiring corneal and limbal transplants at high risk were examined in a study by Sloper CM et al. (2001). According to this study, 23 grafts from 17 individuals were treated with tacrolimus, with a mean optimal dose of 4.4 mg daily for patients with corneal and limbal transplants at high risk. None of the patients who received tacrolimus experienced permanent transplant rejection. Of the nine patients who discontinued therapy within two months, 2 experienced reversible rejection, while five grafts remained clear. As for the eight patients who were still receiving treatment, all of them had clear grafts. The rate of graft rejection is decreased by tacrolimus in high-risk keratoplasty individuals [**48**].

The advantages of employing systemic tacrolimus administration in 47 high-risk corneal grafts to avert rejection were documented by Joseph A et al. (2007). The findings have shown that tacrolimus has the potential to be a viable alternative for individuals undergoing high-risk keratoplasty due to its overall safety and efficacy in prolonging the survival of grafts. However, because of its significant adverse effects, it should only be used with the necessary caution. Before beginning tacrolimus treatment, choosing the appropriate patients, and informing them about the necessity of a cure is crucial [**49**].

## Topical tacrolimus in other anterior segment inflammatory disorders

Several trials were conducted to assess the efficacy of tacrolimus in managing immune-mediated uveitis. Tacrolimus could be utilized to lessen inflammatory activity in uveitis patients because it inhibits T lymphocytes, thereby blocking the inflammatory cytokines release [**8**]. In animal models of uveitis, the topical form of tacrolimus has been shown to prevent endotoxin-induced uveitis and autoimmune uveitis [**50**,**51**]. Tacrolimus is a hydrophobic molecule with a molecular weight of 822 g/mol, which causes limited penetration into the corneal epithelium and solubility in the aqueous humor [**51**]. There is evidence that nano-carrier-based drug delivery technology enhances corneal penetration and retention. Animal studies also found that tacrolimus injections intravitreally suppressed endotoxin-induced uveitis. The results of these studies have led to the assumption that tacrolimus may serve as an effective treatment for patients suffering from uveitis [**52**]. The findings of this research have led to the conclusion that tacrolimus may be a beneficial therapy for uveitis patients.

Following scleral inflammation, T-helper cells rise significantly, with a high ratio of T-helper to T-suppressor. As a result, T lymphocytes play an important role in some forms of scleritis. There is not much evidence supporting the use of tacrolimus to treat scleritis. Considering that tacrolimus suppresses T cells, it could mitigate inflammation in certain types of scleritis. According to Young et al. (2005), refractory surgically produced necrotizing scleritis was treated effectively with systemic tacrolimus [**53**]. Miyazaki et al. (2008) found that topical tacrolimus ointment significantly decreased scleral inflammation in two sclerokeratitis patients and had an additional therapeutic benefit when used with topical steroids [**54**]. Similarly, in 2013, Lee et al. showed that topically administered tacrolimus can control recurrent scleritis, reduce steroid use, and decrease the recurrence of inflammatory reactions [**55**]. Hence topical tacrolimus could be utilized to treat specific types of scleritis, notably for steroid responders, and in addition to standard medicines.

A retrospective study investigated the role of topical tacrolimus in corneal subepithelial infiltrates secondary to adenoviral keratoconjunctivitis management [**56**]. Fifty-four eyes (63.5%) and 31 (36.5%) received tacrolimus 0.03% eye drops twice daily and tacrolimus 0.02% ointment once daily. Subepithelial infiltrates were decreased in number and size in 62.4% of cases, and these filtrates were eliminated in 31.8% of cases. Following tacrolimus therapy, the patient’s visual acuity improved significantly. Tolerability was greater in the group receiving eye drops than in the group receiving topical tacrolimus as ointment. As an outcome, it was established that topical tacrolimus is an effective and safe therapeutic option for subepithelial infiltrates caused by adenoviral keratoconjunctivitis [**56**].

Garcia DP et al., (2011) documented the utilization of a topical ointment called “Protopic” in a 32-year-old male who was suffering from persistent atopic keratoconjunctivitis [**37**]. The patient had previously undergone various treatments, all of which were unsuccessful. His symptoms were significantly reduced and the papillae size was reduced, following two months of using tacrolimus 0.03% ointment twice daily. After undergoing treatment for eight months, there were no signs of remission, and the patient remained asymptomatic [**37**].

Sakarya Y et al. (2012) reported a dramatic improvement in symptoms during the first course of therapy with “Protopic; Astellas Pharma” dermatologic ointment for a refractory atopic blepharoconjunctivitis in a 73-year-old who was unresponsive to conventional corticosteroid therapy. Therapy lasted for a year and no drug-induced side effects were noted when using the tacrolimus ointment. In summary, topical tacrolimus 0.03% dermal ointment seems to be a promising option for atopic keratoconjunctivitis unresponsive to conventional therapy [**57**].

Shoughy SS et al (2020) evaluated the therapeutic role of topical tacrolimus 0.02% eye drops in Thygeson superficial punctate keratitis management. The treatment duration ranged from 1 to 42 weeks. After three days of treatment, the response was observed, with all patients tolerating it well. Topical tacrolimus 0.02% can safely and effectively reduce the ocular surface inflammation in Thygeson superficial punctate keratitis that does not respond to conventional therapy [**58**].

## Conclusion

Topical tacrolimus is currently used to treat ocular inflammation and allergic and immunological diseases of the eye at concentrations ranging from 0.005%-0.1%, as eye drops, and/or ointment, twice daily for an unlimited period, since it is well-tolerated and successful in steroid-resistant refractory ocular inflammatory conditions treatment of individuals. It is a safe, highly effective, and non-invasive long-term use method of treating patients with chronic anterior segment disorders and refractory conditions. It is also considered a preferred initial treatment for patients with ocular inflammation. Some restrictions are off-label and eye drops formulations that could reduce ocular burning complaints have not yet found a better way. However, due to its significant negative effects, it should be used with caution. It is crucial to properly choose and educate the patient before starting tacrolimus medication.

**Conflict of Interest Statement**

The author has no conflicts of interest to declare.

**Acknowledgments**

None.

**Source of Funding**

Not applicable. 

**Disclosures**

None.
